# What Is the Matter with Me, and What Shall I Do?

**Published:** 1888-05

**Authors:** George C. Spearman

**Affiliations:** Pennington, Ga.


### Southern Medical Record.

A MONTHLY JOURNAL OF PRACTICAL MEDICINE.

☞All Communications must be addressed to R. C. WORD, M. D., Managing Editor.

"*Quicquid Præcipies Esto Brevis.*"

VOL. XVIII. ATLANTA, GA., MAY, 1888. No. 5.

### ORIGINAL AND SELECTED ARTICLES.

### WHAT IS THE MATTER WITH ME, AND WHAT SHALL I DO?

BY GEORGE C. SPEARMAN, M.D., OF PENNINGTON, GA.

I report my case for the purpose of asking the profession to give me the correct diagnosis of the case, and what the best treatment will be, and whether or not there is any chance for recovery.

I was forty-one years of age when I was first attacked, which was in June, 1884. Was in my usual health; able to do a good day's riding, or could plough, if necessary, all day long and visit at night to see my patients. I have had dyspepsia ever since I was twenty years old at times, but could always eat enough to keep up and feel "all right." I was stooping down to pick up a buggy spring, and as I was in a little more than half bent position there was a catch came in my back at the junction of sacrum with lumbar vertebra, so that I could not for a time walk without assistance. This kept me on my bed about four weeks, when I got so that I could walk a little with the aid of two sticks. I was then sitting on a chair with my right arm resting on the back of a chair reading my paper, when all at once the sharp catch in my back came again and I could not walk to the bed without two assistants to hold me up and ease me along as best they could. I then remained on my bed for about three months. I could not sit up to eat my meals on account of not being able to bear the weight of my body on the painful spot at the junction of sacrum and vertebra. During these three months the pain extended down the right hip and thigh in the course of the sciatic nerve as far as the hollow of knee. When I got so that I could get out of the house, say about four months after, it extended down the left hip and thigh like the other. I have never had anything like paralysis. I have had a kind of formication in both legs from just above the inner ankle nearly up to my knee, only on the inner side of leg, but that all disappeared nearly eight months ago. There still remains some stiffness and a feeling of heaviness in the calves of my legs. I have frequently had these catches in my back at the same spot when I would attempt to lift something, but after putting on a porous plaster, and having to keep my bed for about eight days, I could get out and be "all right" for anything that my business would call for. I suppose that I have had about eight of these catches in my back from the close of the last year up to the time of this last attack. My general health has given away considerably since I was attacked this last time. My nervous system generally has given away, as well as my digestion and assimilation.

I will give my feelings and symptoms at present the best I can, also the treatment I have had and my diagnosis. The diagnosis is sciatica, which has resulted, with dyspepsia, in neurasthenia or nervous exhaustion. Theine has been used hypodermically; other remedies have been used from time to time—strychnia, iron, quinine, nux vomica, salicylate sodium, cohosh, muriatic acid, iodine iodide potassium, ergot—but my stomach would not bear large doses of anything. Have used counter-iritation in the form of liniments and pustulating liniments. There has never been but little tenderness on pressure, no heat, redness, or swelling at any time. Hot applications will ease the pain, and no matter how bad it may be hurting me, if I lie down and get warm, it will get easy and remain so until I get up and begin to walk about or stand up too long. If I walk too much, say half mile, or stand still half an hour, or attempt to stoop or lean too far backwards, it gives me great pain, but if I then lie down it will get easy again. It never hurts me at night as rheumatism does. I have a great dread of something bad going to happen that I can't throw off. I am very despondent, losing confidence in myself and everything else. I have a great desire to be doing something, but can't get up courage enough to make the attempt for fear of pain. I used to be good humored, but have become very irritable, nothing goes right. Have lost all hope of recovery, which may perhaps be imagination. I feel at times that all my friends had deserted me; then again I feel away up in the skies, and that every body was my friend. I know that all this is nervousness and weakness, but it seems that I have not the moral courage to overcome it. I have lost all my venereal desire over a year ago. I don't know that this will assist in your diagnosis or treatment, or be of any assistance to you, but think it well to mention it. I have always been temperate, never took a drink of whiskey in my life. I have always been moderate in my venereal desires; there is no trouble about erections, only that I have little or no desire. My greatest trouble is pain in my back not letting me stoop, reach or exercise, and my dyspepsia. My average weight was one hundred and twenty-five to one hundred and twenty-eight, but since I have been afflicted my weight fell to one hundred and three, but I weigh at this time one hundred and sixteen pounds.

I am also troubled at times with the same kind of catch in my neck, about axis, that will not let me turn my head to the right or left, or look up or down. The spells will last about one week and then get better. There was none of this until about one year ago. It and my back is always worse if I happen to eat anything that unusually disagrees with my stomach. My bowels are constipated and I have to take cascara regularly. They are often distended with gas. Sometimes my stomach is nauseated; but I am never troubled with pain in stomach or bowels. My memory is not good as it used to be, and am not capable of much thought. I sleep well at night, generally, and feel better every morning than I do the balance of the day. If I sit down long and get up to walk it feels like there was something in my back at the top of the sacrum like a splinter or the point of a knife that will stick, and when I start to walk it will stick in first one side of sacrum and then the other, extending down as far as the back part of trochanters, but after I make a few careful steps it gets so that I can get along without much pain unless I try to go too far, and if I do, it will get to aching so bad all over the glutial region, from the crest of ilium and top of sacrum down to trochanters, that I will be compelled to either sit down or lie down. I can't sit on a very low seat without a great deal of pain.

I believe I have about given all that is of importance, and now if any of the profession can give me any light as to diagnosis and treatment, I will certainly accept it as a great favor. If I am not clear enough in my inquiry and description, will be glad to answer any question that will be asked through the RECORD or privately, and will pay all expenses. Hope the profession will make due allowance for errors and avoidance of technicalties, as I am hardly able to write at all.

